# Prevalence and determinants of chronic kidney disease in community-dwelling elderly by various estimating equations

**DOI:** 10.1186/1471-2458-12-343

**Published:** 2012-05-10

**Authors:** Dietrich Rothenbacher, Jochen Klenk, Michael Denkinger, Mahir Karakas, Thorsten Nikolaus, Richard Peter, Wolfgang Koenig

**Affiliations:** 1 Institute of Epidemiology and Medical Biometry, Ulm University, Ulm, Helmholtzstr 22, D-89081, Germany; 2 Agaplesion Bethesda Clinic Ulm, Ulm, Germany; 3 Department of Internal Medicine II-Cardiology, University of Ulm Medical Centre, Ulm, Germany

**Keywords:** Elderly, Chronic kidney disease, Population-based study, Estimating equations, Risk factors

## Abstract

**Background:**

Chronic kidney disease (CKD) represents a global public health problem. Few data exist in the elderly. The objective of the current study is to estimate the prevalence of CKD by means of various established and new equations and to identify the main determinants of CKD in elderly.

**Methods:**

The ActiFE Ulm (Activity and Function in the Elderly in Ulm) study is a population-based cohort study in people of 65 years and older. Kidney function was assessed by means of estimated glomerular filtration rate (eGFR) based on two creatinine- (Cr-; MDRD, CKD-EPI) and one cystatin C - (CysC-) based method. The relationship between various potential risk factors and CKD was quantified using unconditional logistic regression.

**Results:**

A total of 1471 subjects were in the final analysis (mean age 75.6 years, SD 6.56). Overall, prevalence of CKD (eGFR < 60 mL/min/1.73 m^2^) was 34.3% by MDRD, 33.0% by CKD-EPI, and 14.6% by the CysC-based eGFR. All eGFRs showed statistically significant correlations with C-reactive protein, uric acid, as well as with lipid values. In multivariable analysis age was clearly related to prevalence of CKD and the risks were highest with the CysC-based equation. Females had a higher risk for CKD stages 3–5 with MDRD (OR 1.63; 95% CI: 1.23–2.16) whereas the OR was 1.23 (95% CI 0.92–1.65) with the CKD-Epi and OR = 0.89 (95% CI 0.58–1.34) with the CysC-based equation after multivariable adjustment. Although the cystatin C based definition of CKD resulted in a lower prevalence compared to the creatinine based ones, other measures of renal damage such as albuminuria were more prevalent in those defined by CysC-eGFR.

**Conclusions:**

Prevalence of CKD is very variable based on the used estimating equation. More work is needed to evaluate the various estimating equations especially in elderly before we are able to assess the practical consequences of the observed differences.

## Background

Chronic kidney disease (CKD) represents a global public health problem and affects a large proportion of the adult population worldwide [1,2]. CKD has a complicated relationship with diabetes and hypertension and other associated diseases, and it is an independent risk factor for cardiovascular diseases (CVD) as well as for all cause mortality [1]. Outcomes of CKD include not only progression to end-stage renal disease (ESRD) but also complications such as hypertension, malnutrition, anaemia, bone disease and a decreased quality of life [3,4]. CKD is also a proposed risk factor for adverse outcomes in other chronic diseases such as infections and various cancers.

In the meantime a paradigm shift has occurred. Early, subclinical CKD (and not only ESRD) has been associated with a large burden of disease and mortality [5]. This finding is clinically important, because early detection and treatment of CKD can prevent or delay the progression of CKD to ESRD and to other more common, but still severe complications related to CVD and to potential side effects or overdosing of medication.

Since the Kidney Disease Outcome Quality Initiation (K/DOQI) clinical practice guideline for definition and classification of CKD have been published and updated [5,6], more epidemiologic data about prevalence of CKD in the general population are available. However, few studies focused on risk factors for early stages of CKD among elderly and used the different suggested estimating equations of glomerular filtration rate (eGFR) to assess renal function in clinical practice.

The objective of the current study is to estimate the prevalence of CKD by means of Cr- and CysC- based estimating equations and to identify the main determinants of CKD in a large population-based group of non-institutionalized elderly subjects.

## Methods

### Ethics statement

The study was approved by the ethical committee of Ulm University. All participants provided written informed consent.

### Study population

The ActiFE Ulm (Activity and Function in the Elderly in Ulm) study is a population-based cohort study in subjects aged 65 years and older, located in Ulm and adjacent regions in Southern Germany. Based on data from the local registry office a random-sample of 7,624 non-institutionalized inhabitants was contacted by mail and asked for participation. Exclusion criteria were severe deficits in cognition, vision or hearing that precluded the accomplishment of most assessments or serious German language difficulties. Between March 2009 and April 2010, 1,506 eligible individuals agreed to participate and underwent the baseline assessments (participation rate: 19.8%). Further details are described elsewhere [7,8].

### Data collection

Participants satisfying the inclusion criteria were contacted by a field worker to make an individual appointment for an interview at home. Participants who did not want to be visited in their home were given the alternative to meet the interviewer in a designated room located at the Bethesda Geriatric Clinic, Ulm.

In total there were three visits incorporated in the ActiFE Ulm study, all to be completed within seven days. The first and last visits were conducted by a study nurse, the visit in between by a physician who conducted a physical exam. During the first visit the interviewer obtained informed consent from the participant, provided information about the study procedure and conducted the first half of the baseline interview. The second visit was conducted by a physician. In the last visit the interviewer accomplished the second half of the baseline interview. Briefly, the core questionnaires included questions on socio-demographic characteristics, diagnosis and related respiratory symptoms of asthma and COPD, physical functioning and activity, comorbidity (“has a doctor ever told you…”), exposures and potential risk factors related to asthma, COPD and physical activity, clinical management (treatment and self management issues), accessibility and use of health services.

If participants had separately agreed, blood was collected during the second visit. Participants who were successfully instructed on visit one, also provided mid-stream urine (morning void).

### Laboratory methods

Blood at baseline was drawn in fasting state under standardized conditions. Serum creatinine (Cr) was measured by the kinetic Jaffe method (inter-assay CV 1.2–3.0%) on a IMDS-traceable reference standard. Serum cystatin C (CysC) was measured by immunonephelometry on a Behring Nephelometer II (inter-assay CV 2.9–3.2%). C-reactive protein (CRP) was determined by a high-sensitivity assay on the same device (inter-assay CV 5.2–6.4%). Urinary albumin was measured by immunonephelometry assay (inter-assay CV 2.8%). Blood lipid measurements and other measurements were done by routine methods. All markers were measured in a blinded fashion.

### Assessment of chronic kidney disease

Kidney function was assessed by means of eGFR based on the three estimating equations as described below:

*Cr-eGFR according to the Simplified Modification of Diet in Renal Disease (MDRD) equation*[9]: $\text{Cr}-\text{eGFR}\left(\text{MDRD}\right)=175\times {\left(\text{Cr}\right)}^{-1.154}\times {\left(\text{age}\right)}^{-0.203}\times \left(0.742\;\text{if female}\right)\times \left(1.21\;\text{if African American}\right)\text{.}$

*eGFRcrea according to the Chronic Kidney Disease Epidemiology Collaboration (CKD-EPI) equation*[10]: $\text{eGFR}=141\times \text{min}{\left(\text{Cr}/\text{k},1\right)}^{\text{a}}\times \text{max}\;\left(\text{Cr}/\text{k},1\right){-}^{1.209}\times {\left(0.993\right)}^{\text{age}}\times \left(1.018\;\text{if female}\right)\times \left(1.159\;\text{if black}\right)$ where k is 0.7 for females and 0.9 for males, a is −0.329 for females and −0.411 for males, min indicates the minimum of Cr/k or 1, and max indicates the maximum of Cr/k or 1.

*Cystatin C-based eGFR according to CKD-EPI collaboration*[11,12]: $\text{CysC}-\text{eGFR}\left(\text{CKD}-\text{EPI}\right)=127.7\times {\left(\text{CysC}\right)}^{-1.17}\times {\text{age}}^{-0.13}\times \left(0.91\;\text{if female}\right)\times \left(1.06\;\text{if black}\right)\text{.}$

CKD stage 3–5 was defined as eGFR of less than 60 mL/min/1.73 m^2^[13]. In equations, Cr is given in mg/dL, CysC in mg/L, age in years, weight in kg, and eGFR in mL/min/1.73 m^2^.

Renal damage was defined as albumin-to-creatinine ratio (ACR) in spot urine sample (microalbuminuria ACR 30 to < 300 mg/g, macroalbuminuria ≥ 300 mg/g).

### Statistical analysis

Descriptive statistics were calculated to describe the main characteristics of the study population. In addition prevalence of CKD was calculated according to the various eGFR estimating equations, stratified by age and gender. Correlations between various renal function markers, eGFR and biochemical markers were calculated by the non-parametric partial correlation coefficient after adjustment for age and gender. The relationship between various potential risk factors and CKD was quantified using unconditional logistic regression. Mainly factors which were described as potential risk factors in the literature were considered. Odds ratios (OR) and 95% confidence intervals (CI) were estimated in crude and adjusted analyses. All analyses were performed using SAS 9.2.

## Results

Overall 1471 subjects aged 65 and older with complete data on creatinine, cystatin C and albumin were included in the final analysis (mean age 75.6 years, SD 6.56) (Table 1). More males were included than females (56.8% versus 43.2%). Most of the subjects were married (65.5%) and had a school education of 9 years or less (58.6%). The mean body mass index was 27.6 kg/m^2^ and 24.2% were obese. More than half had a history of hypertension, 8.6% had myocardial infarction, 14.5% a history of heart failure, and 13.3% reported a physician-diagnosed history of diabetes. Only 2.9% reported a history of CKD.

**Table 1:** Characteristics of study population (n = 1471)

**Age, mean (SD)**	**years**	**75.6 (6.56)**
n (%)	65-69	351 (23.9)
	70-79	673 (45.8)
	≥80	447 (30.4)
Gender, n (%)	male	836 (56.8)
	female	635 (43.2)
Family status, n (%)	married	960 (65.5)
	widowed	362 (24.7)
Duration of school education,	≤9 yrs	849 (58.6)
n (%)	10-11 yrs	334 (23.1)
	≥12 yrs	266 (18.4)
Alcohol consumption, n (%)	daily	448 (31.0)
	several time per week or month	741 (51.3)
	<1 time per month	243 (16.8)
	never	12 (0.8)
Smoking status, n (%)	smoker	136 (9.3)
	former smoker	601 (41.0)
	never-smoker	729 (49.7)
Body Mass Index, mean (SD)	kg/m^2^	27.6 (4.16)
	≥30 kg/m^2^, n (%)	356 (24.2)
History of co-morbidity n (%)	hypertension	784 (53.3)
	myocardial infarct	127 (8.6)
	heart failure	213 (14.5)
	chronic kidney disease	43 (2.9)
	diabetes	197 (13.4)
Creatinine ^a^	mg/dL	0.98 (0.87; 1.14)
Cystatin C ^a^	mg/L	0.88 (0.77; 1.03)
C-reactive protein ^a^	mg/L	1.70 (0.89; 3.70)

^a^median (interquartile range, Q1, Q3).

**Figure 1:**
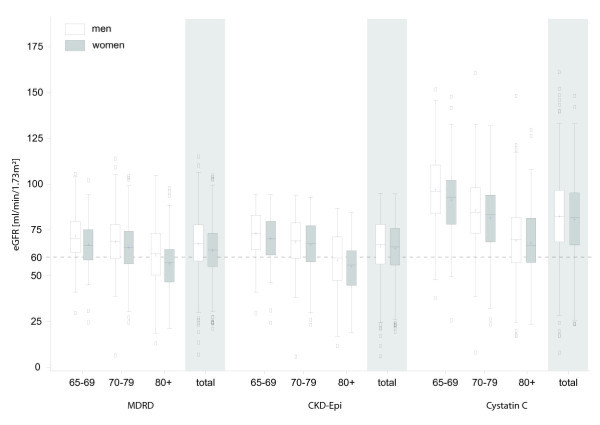
Boxplots of eGFR based on different estimating equations for men and women in different age groups (values lower than 60 ml/min/1.73 m2 indicating chronic kidney disease stages 3–5; + denoting mean values).

The mean (SD) of eGFR was 65.2 (14.6), 65.5 (14.9), and 81.7 (21.8) mL/min/1.73 m^2^ respectively, based on MDRD, CKD-EPI and CysC estimating equation. Figure 1 shows the distribution of eGFR in a box plot according to males and females and in the various age categories. In general, values were much higher when the CysC based equations were applied.

Table 2 shows the prevalence of the various stages of CKD. By means of the MDRD, the CKD-EPI, and the CysC-based eGFR, 61.2%, 62.5%, and 79.3%, respectively, had no kidney damage with normal or mildly decreased eGFR. Notably, especially among women these numbers were especially different ((MDRD, 58.0%, CKD-EPI 62.4%, CysC-based CKD 82.2%).

**Table 2:** Prevalence of stages of chronic kidney disease according to the various estimating equations

**Marker**	**Stage of**	**Description**	**eGFR**^**a**^	**Men**	**Women**	**Total**
	**CKD**			**N (%)**	**N (%)**	**N (%)**
MDRD	-	No kidney damage with normal or mildly decreased eGFR	≥60	532 (63.6)	368 (58.0)	900 (61.2)
	1	Kidney damage with normal eGFR	≥90	3 (0.4)	1 (0.2)	4 (0.3)
	2	Kidney damage with mildly decreased eGFR	60–89	46 (5.5)	17 (2.7)	63 (4.3)
	3	Moderately decreased eGFR	30–59	245 (29.3)	239 (37.6)	484 (32.9)
	4/5	Severely decreased eGFR, kidney failure	<30	10 (1.2)	10 (1.6)	20 (1.4)
CKD-EPI	-	No kidney damage with normal or mildly decreased eGFR	≥60	523 (62.6)	396 (62.4)	919 (62.5)
	1	Kidney damage with normal eGFR	≥90	2 (0.2)	1 (0.2)	3 (0.2)
	2	Kidney damage with mildly decreased eGFR	60–89	46 (5.5)	17 (2.7)	63 (4.3)
	3	Moderately decreased eGFR	30–59	253 (30.3)	209 (32.9)	462 (31.4)
	4/5	Severely decreased eGFR, kidney failure	<30	12 (1.4)	12 (1.9)	24 (1.6)
Cystatin-C based eGFR	-	No kidney damage with normal or mildly decreased eGFR	≥60	645 (77.2)	522 (82.2)	1167 (79.3)
	1	Kidney damage with normal eGFR	≥90	18 (2.2)	4 (0.6)	22 (1.5)
	2	Kidney damage with mildly decreased eGFR	60–89	53 (6.3)	15 (2.4)	68 (4.6)
	3	Moderately decreased eGFR	30–59	113 (13.5)	89 (14.0)	202 (13.7)
	4/5	Severely decreased eGFR, kidney failure	<30	7 (0.8)	5 (0.8)	12 (0.8)

^a^ = mL/min per 1.73 m^2^.

Table 3 shows the prevalence of CKD by means of different estimating equations. Overall, prevalence of CKD stage 3–5 was 34.3% by MDRD, 33.0% by CKD-EPI, and 14.6% by the CysC based estimating equations. 9.9% had a micro- or macroalbuminuria. On the basis of the creatinine-based eGFRs women had a higher prevalence compared to men, this was most pronounced with the MDRD; the highest prevalence was reached in the oldest age categories, reaching 61.4% in women aged 80 and over based on the CKD-EPI eGFR. In contrast, the CysC based eGFR showed a higher prevalence in men when compared to women and the increase with age was less steep, reaching a maximum of 32.3% in the category women aged 80 and over.

**Table 3:** **Prevalence of chronic kidney disease stages 3–5 (eGFR <60 mL/min per 1.73 m**^**2**^**) in various age groups and according to gender based on different estimating equations**

**Marker**	**Age group**	**Men**	**Women**	**Total**
		**N (%)**	**N (%)**	**N (%)**
MDRD	65–69	26 (14.5)	50 (29.1)	76 (21.7)
	70–79	101 (27.5)	105 (34.4)	206 (30.6)
	80+	128 (44.3)	94 (59.5)	222 (49.7)
	Overall	255 (30.5)	249 (39.2)	504 (34.3)
CKD-EPI	65–69	21 (11.7)	34 (19.8)	55 (15.7)
	70–79	98 (26.6)	90 (29.5)	188 (27.9)
	80+	146 (50.5)	97 (61.4)	243 (54.4)
	Overall	265 (31.7)	221 (34.8)	486 (33.0)
Cystatin-C based eGFR	65–69	4 (2.2)	7 (4.1)	11 (3.1)
	70–79	30 (8.2)	36 (11.8)	66 (9.8)
	80+	86 (29.8)	51 (32.3)	137 (30.7)
	Overall	120 (14.4)	94 (14.8)	214 (14.6)
Albumin creatinine ratio (ACR) > 30 mg/g	65–69	17 (9.5)	7 (4.1)	24 (6.8)
	70–79	43 (11.7)	12 (3.9)	55 (8.2)
	80+	49 (17.0)	17 (10.8)	66 (14.8)
	Overall	109 (13.0)	36 (5.7)	145 (9.9)

Table 4 shows the age and gender adjusted partial correlation of the various renal function markers and eGFRs with various biochemical markers. The correlation between Cr and CysC was 0.61 (p < 0.001). The correlation of Cr and CysC with albumin in the urine was 0.08 (p = <0.001) and 0.13 (p < 0.001), respectively. Both Cr-based eGFRs showed no correlation with ACR, whereas the CysC based eGFR did (r = −0.07, p = <0.001). All eGFRs showed statistically significant correlations with markers of inflammation (CRP, WBC), uric acid, as well as with lipid values. Age- and gender adjusted associations of Cr, CysC with haemoglobin were negative and with MDRD, CKD-Epi and CysC-based eGFR positive, indicating that higher eGFR levels were associated with higher haemoglobin.

**Table 4:** Association of different renal function parameters and estimation equations with biochemical markers after adjustment for age and gender (spearman partial correlation coefficient and p-value)

	**CysC (mg/L)**	**MDRD eGFR**	**CKD-EPI eGFR**	**CysC- eGFR**	**ACR**	**CRP (mg/L)**	**Uric acid (μmol/L)**	**Cholesterol (mmol/L)**	**HDL (mmol/L)**	**LDL (mmol/L)**	**WBC (Giga/L)**	**Hemoglobin (g/dL)**
Creatinine (mg/dL)	0.61	−0.96	−0.96	−0.60	−0.00	0.13	0.44	−0.07	−0.18	−0.06	0.07	−0.05
	<0.001	<0.001	<0.001	<0.001	0.89	<0.001	<0.001	0.005	<0.001	0.03	0.007	0.08
CysC (mg/L)		−0.60	−0.60	−1.00	0.07	0.24	0.39	−0.10	−0.24	−0.05	0.10	−0.08
		<0.001	<0.001	<0.001	0.006	<0.001	<0.001	<0.001	<0.001	0.04	<0.001	0.003
MDRD eGFR			1.00	0.60	0.00	−0.14	−0.41	0.07	0.16	0.06	−0.06	0.05
			<0.001	<0.001	0.91	<0.001	<0.001	0.011	<0.001	0.03	0.02	0.04
CKD-Epi eGFR				0.60	0.02	−0.13	−0.41	0.07	0.16	0.06	−0.06	0.05
				<0.001	0.95	<0.001	<0.001	0.01	<0.001	0.03	0.02	0.04
Cystatin-C based eGFR					−0.07	−0.25	−0.38	0.09	0.24	0.05	−0.10	0.08
					0.005	<0.001	<0.001	<0.001	<0.001	0.04	<0.001	0.001
ACR (urine)						0.10	0.03	−0.04	−0.03	−0.05	0.06	−0.07
						<0.001	0.23	0.14	0.27	0.08	0.03	0.01

*Abbreviations*: ACR = Albumine-creatinine ratio (mg/g), CysC = cystatine C, MDRD = Modification of Diet in Renal Disease Study, eGFR = estimated glomerular filtration rate.

(mL/min/1.73 m^2^), CKD-Epi = Chronic Kidney Disease Epidemiology Collaboration, CRP = C-reactive protein, HDL = high-density lipoprotein, LDL = low density lipoprotein,WBC = white blood cell count.

Table 5 shows the prevalence of albuminuria, an established renal damage marker, with CKD based on the different estimating equations in the subset of elderly. The proportions of micro- and marcoalbuminuria were quite similar among MDRD and CKD-Epi, they were higher among the CysC based eGFR.

**Table 5:** **Prevalence of micro- and macroalbuminuria in subjects with and without chronic kidney disease stages 3–5 (eGFR <60 mL/min per 1.73 m**^**2**^**) based on different estimating equations**

	**Chronic kidney disease ((eGFR <60 mL/min per 1.73 m**^**2**^**)**					
	**MDRD GFR**		**CKD-Epi**		**CsyC-based**	
	no	yes	no	yes	no	yes
Normal, N (%)	900 (93.1)	426 (84.5)	919 (93.3)	407 (83.7)	1167 (92.8)	159 (74.3)
Microalbuminuria, N (%)	60 (6.2)	65 (12.9)	59 (6.0)	66 (13.6)	79 (6.3)	46 (21.5)
Macroalbuminuria, N (%)	7 (0.7)	13 (2.6)	7 (0.7)	13 (2.7)	11 (0.9)	9 (4.2)
Total, N (%)	967 (100.0)	504 (100.0)	985 (100.0)	486 (100.0)	1257 (100.0)	214 (100.0)

Microalbuminuria ACR 30 to < 300 mg/g, macroalbuminuria ACR ≥ 300 mg/g.

Table 6 presents the association of various sociodemographic and medical characteristics with prevalence of CKD stage 3–5. Age was clearly related to prevalence of CKD and risks were highest in the CysC-based equation. Females had a higher risk for CKD stage 3–5 with MDRD (OR 1.63; 95% CI: 1.23–2.16) whereas the OR was 1.23 (95% CI 0.92–1.65) with the CKD-Epi and OR = 0.89 (95% CI 0.58–1.34) with the CysC-based equation after multivariable adjustment.

**Table 6:** **Association of various factors with chronic kidney disease (eGFR <60 mL/min/1.73 m**^**2**^**) based on different estimating equations**

		**MDRD GFR**		**CKD-Epi**		**CsyC-based CKD**	
		**crude**	**adjusted**^**a**^	**crude**	**adjusted**^**a**^	**crude**	**adjusted**^**a**^
Age	65–69	1.00 ^(referent)^	1.00^(referent)^	1.00^(referent)^	1.00^(referent)^	1.00^(referent)^	1.00^(referent)^
	70–79	1.60 (1.18; 2.16)	1.52 (1.11; 2.10)	2.09 (1.49; 2.91)	1.98 (1.40; 2.81)	3.36 (1.75; 6.45)	3.30 (1.64; 6.67)
	≥80	3.57 (2.61; 4.89)	3.33 (2.37; 4.74)	6.41 (4.55; 9.03)	6.01 (4.14; 8.73)	13.66 (7.25; 25.73)	13.94 (6.95; 27.98)
Gender	female	1.47 (1.18; 1.83)	1.63 (1.23; 2.16)	1.15 (0.92; 1.43)	1.23 (0.92; 1.65)	1.04 (0.77; 1.39)	0.89 (0.58; 1.34)
Family status	Married	1.00^(referent)^	1.00^(referent)^	1.00^(referent)^	1.00^(referent)^	1.00^(referent)^	1.00^(referent)^
	widowed	1.94 (1.51; 2.49)	1.24 (0.93; 1.65)	2.09 (1.63; 2.69)	1.29 (0.96; 1.74)	2.11 (1.54; 2.90)	1.17 (0.79; 1.74)
	other	1.29 (0.89; 1.86)	1.12 (0.75; 1.67)	1.40 (0.97; 2.03)	1.33 (0.88; 2.00)	1.29 (0.78; 2.14)	1.34 (0.75; 2.38)
Duration of	≤9 yrs	1.00^(referent)^	1.00^(referent)^	1.00^(referent)^	1.00^(referent)^	1.00^(referent)^	1.00^(referent)^
school education	10–11 yrs	1.01 (0.77; 1.31)	1.11 (0.83; 1.47)	1.00 (0.76; 1.31)	1.13 (0.84; 1.52)	1.02 (0.72; 1.46)	1.17 (0.78; 1.75)
	≥12 yrs	0.96 (0.72; 1.29)	1.18 (0.86; 1.63)	0.93 (0.69; 1.25)	1.11 (0.80; 1.54)	0.72 (0.47; 1.10)	0.85 (0.53; 1.37)
Alcohol consumption	daily	0.49 (0.36; 0.68)	0.69 (0.48; 1.01)	0.54 (0.39; 0.75)	0.66 (0.45; 0.97)	0.43 (0.29; 0.64)	0.40 (0.24; 0.66)
	several time per week or per month	0.54 (0.40; 0.72)	0.72 (0.52; 1.00)	0.55 (0.41; 0.74)	0.70 (0.50; 0.98)	0.37 (0.26; 0.54)	0.39 (0.25; 0.60)
	<1 time per month	1.00^(referent)^	1.00^(referent)^	1.00^(referent)^	1.00^(referent)^	1.00^(referent)^	1.00^(referent)^
	never	0.81 (0.25; 2.62)	0.57 (0.17; 1.95)	1.25 (0.39; 3.99)	0.93 (0.28; 3.14)	0.97 (0.26; 3.71)	0.68 (0.16; 2.91)
Smoking status	smoker	0.56 (0.37; 0.86)	0.73 (0.46; 1.16)	0.61 (0.40; 0.94)	0.77 (0.48; 1.24)	0.85 (0.50; 1.45)	1.11 (0.58; 2.12)
	former smoker	0.91 (0.73; 1.14)	1.04 (0.79; 1.35)	0.96 (0.77; 1.21)	0.99 (0.75; 1.31)	0.89 (0.66; 1.21)	0.83 (0.56; 1.22)
	never-smoker	1.00^(referent)^	1.00^(referent)^	1.00^(referent)^	1.00^(referent)^	1.00^(referent)^	1.00^(referent)^
Body Mass Index	≥30 kg/m^2^, n (%)	1.57 (1.22; 2.00)	1.48 (1.13; 1.94)	1.51 (1.18; 1.93)	1.52 (1.15; 2.01)	1.87 (1.37; 2.56)	1.98 (1.37; 2.85)
History of Co-morbidity,	hypertension	1.60 (1.29; 1.99)	1.28 (1.00; 1.63)	1.59 (1.27; 1.98)	1.23 (0.96; 1.59)	2.08 (1.53; 2.84)	1.54 (1.07; 2.21)
	myocardial infarct	2.40 (1.66; 3.46)	2.18 (1.45; 3.26)	2.84 (1.96; 4.10)	2.40 (1.59; 3.62)	3.23 (2.15; 4.84)	2.22 (1.39; 3.56)
	heart failure	1.49 (1.11; 2.01)	1.12 (0.80; 1.55)	1.45 (1.08; 1.96)	1.03 (0.74; 1.45)	2.41 (1.70; 3.41)	1.90 (1.27; 2.84)
	diabetes	1.59 (1.17; 2.15)	1.37 (0.98; 1.92)	1.43 (1.09; 1.94)	1.14 (0.80; 1.62)	1.67 (1.14; 2.44)	1.14 (0.74; 1.77)

^a^ Multivariate logistic regression model and 95% confidence interval (95% CI) adjusted for gender, age, family status, duration of school education, alcohol consumption, smoking status, adipositas and history of co-morbidity (hypertension, myocardial infarction, heart failure, diabetes).

Alcohol consumption showed also a negative association with prevalence of CKD and remained statistically significant after full adjustment for all estimating equations. Furthermore, similar associations were seen with manifest obesity, partly hypertension and subjects with a history of myocardial infarction with all three estimating equations, and with heart failure with the CysC-based CKD definition.

## Discussion

In this large population based study involving 1471 subjects aged 65 to 91 years and randomly selected from the general population in the area of Ulm (Germany) prevalence of chronic kidney disease stage 3–5 varied considerably between 14.6% on a CysC-based estimating equation to 33.0% and 34.3% based on CKD-EPI and MDRD estimating equation, respectively. A steep increase with age was found with all three equations. Although the cystatin C based definition of CKD resulted in a lower prevalence compared to the creatinine based ones, other measures of renal damage such as albuminuria were more prevalent in those defined by CysC-eGFR. The association of gender with CKD was quite different, depending on the definition used: women had a higher risk for CKD with the MDRD.

The prevalence of CKD in our elderly population is roughly in line with data from others studies when considering the Cr-based estimating equations [2]. In a different population based study in subjects aged 50–75 years also conducted in Germany and using the MDRD, however, the prevalence was slightly higher in comparable age strata [14]. Prevalence of CKD in the Three-City study including 8705 community-dwelling elderly aged 65 years or older was 13.7% and 12.9% with the MDRD and CKD-EPI equation, and the long-term risk estimates were quite similar with both equations [15]. In a study from the US conducted in octogenarians (mean age 86 years) in which beside CKD-EPI and also two cystatin C based equations were used to define CKD, the patterns were quite similar to our results [16]. In this study of Shastri et al. the CKD-EPI resulted in prevalence of 51.3% and with the one-variable CysC-based equation in a prevalence of 33.0%. In our study, in the respective age category of 80+ years the CKD-EPI lead to a prevalence of 54.4% and the CysC-based equation to 30.7%. In a study from the US conducted in elderly patients aged 65 years and over the prevalence of CKD based on the MDRD was 44% [17].

Notably we found a higher prevalence of CKD in women when using the MDRD estimating equation. It is consistent with results from a study conducted in a population of elderly from another State in Germany [14] and has been a consistent feature in a systematic review [2]. Beside gender differences in physiological factors the lower prevalence may also be a function of the correction factor for females in the Cr-based equations. Men are de facto also at higher risk for ESRD compared to women, which is also indirect evidence for a higher burden of the underlying CKD, which finally may progress to ESRD [18].

In addition it is known that especially the MDRD-based eGFR overestimates renal function in general in elderly subjects [19] and part of the high prevalence may be accountable to it. In a population of volunteers (mean age 61.3 years, SD 8.6) MDRD underestimated eGFR in a healthy population compared to CysC-based ones [20]. Cr-based eGFR may show a large intra- and inter-individual variation due to the dependence of Cr concentration on many factors such as muscle mass, dietary intakes and other diseases [2]. Recently the CKD-EPI formulation had been proposed to work better in the context of epidemiological studies [10]. CKD-EPI seems to misclassify fewer low-risk patients compared to MDRD [21]. Another study reported that compared to MDRD the CKD-EPI leads to higher estimates in young people and to lower estimates in elderly. The authors suggested an age dependent threshold for CKD [22].

We found a stronger association of established risk factors, comorbid conditions, and biochemical markers related more or less to risk for kidney disease with CysC-based definition of CKD. The only exception was uric acid which was stronger for Cr-based eGFR. The association of metabolic risk factors such as HDL and insulin resistance (although the latter was not available in our study) had been described with early impairment of renal function, irrespective of markers of renal damage [23].

Several studies consistently found a better prognostic value of cystatin C or CysC-based estimating equations with all-cause mortality [24,25], CVD [26], and ESRD [24,27]. Adding CysC- to Cr-based measures to improve predictive accuracy was also suggested by some authors [24], however, this approach was not superior in other studies [26]. A meta-analysis also concluded that the diagnostic accuracy for measuring renal function is in favour for CysC when compared to Cr. However, the study included a very heterogeneous patient population and only few elderly [28].

Currently there is a hot debate about the definition of CKD in elderly based on a fixed threshold [29,30]. The argument is that screening will identify many false positives, especially among elderly women, most of them having no evidence of kidney disease. The decrease in kidney function might be due to changes in kidney structure associated with aging [31] and be considered physiological. However, in a collaborative meta-analysis including more than 100,000 subjects from 14 studies the shape between eGFR and risk of all cause- and CVD-mortality was similar in age groups below and above age 65 years and the test for interaction between eGFR and age were not significant in most studies [1].

Although we found a strong association of CKD stage 3–5 with various cardiovascular diseases and risk factors, we found a statistically significant association with history of diabetes only in bivariate analyses, consistently for all three estimating equations. The lack of statistically significant associations with diabetes has also been reported by other investigators [14,22].

When looking on the results the following limitations should be considered. We did not have GFR measurement, so we cannot determine the extent to which either CysC or Cr reflects the glomerular filtration rate as determined by a gold standard (i.e. inulin clearance). However, it is very difficult to obtain true GFR measurements in such a large population. Also, we only used a cross-sectional design, so the temporal association between the described risk factors and CKD cannot be assessed. Furthermore, we only had a single measurement of CKD, whereas usually for definition of CKD two measures within a time period of 3 months should be employed. The latter, however, is a limitation present in most of the cited epidemiological studies, but may indeed cause an increase in prevalence numbers. In addition, we used only one among the many suggested CysC-eGFR equations. Current available data on CysC-eGFR equations are still very limited. Results from this study does not exclude the possibility that CysC-based eGFR overestimates GFR in the elderly. In this case, wide use of this equation may expose this population to drug overdosing and adverse effects. However, it is notably, that the proportion with no signs of renal damage is highest in the group with no CKD based on the CysC-CKD definition. Therefore, further studies should evaluate the CysC-eGFR equations in large diverse populations and further standardization of assays is needed; especially with the Siemens Cystatin C method calibration has changed during the last five years; a downward shift in calibrator had occurred between 2006 and 2009 [32]. This has to be considered if results obtained from older lots are compared to the current study.

## Conclusions

Our study in a large group of elderly shows that prevalence of CKD is very variable based on the estimating equations used and implies that CysC- based assessment of renal function may be more specific and helpful to identify a group of patients that may benefit from a specific intervention. More work is needed to comparatively evaluate the various estimating equations especially in elderly before we are able to assess the practical consequences of the observed differences.

## Abbreviations

CKD: Chronic kidney disease; CKD-EPI: Chronic Kidney Disease Epidemiology (collaboration); CVD: Cardiovascular disease; CysC: Cystatin C; Cr: Creatinine; eGFR: Estimated glomerular filtration rate; ESRD: End stage renal disease.

## Competing interest

The authors declare that they have no competing interests.

## Authors’ contributions

DR had conceived this study and drafted the manuscript. WK and MK organized and coordinated the laboratory measurements and helped to draft the ms. JK performed the statistical analysis. MD, TN, RP participated in the design and conduct, and coordination of the study. All authors read and approved the final manuscript.

## Pre-publication history

The pre-publication history for this paper can be accessed here:

http://www.biomedcentral.com/1471-2458/12/343/prepub
